# The effect of externally generated loading on predictive grip force modulation

**DOI:** 10.1016/j.neulet.2006.10.026

**Published:** 2007-02-27

**Authors:** Alice G. Witney, Daniel M. Wolpert

**Affiliations:** a Department of Zoology, University of Cambridge, Downing Street, Cambridge CB2 3JN, UK; b Department of Engineering, University of Cambridge, Trumpington Street, Cambridge CB2 1PZ, UK

**Keywords:** Predictive models, Grip force

## Abstract

A characteristic of skilled movement is the ability of the CNS to predict the consequences of motor commands. When we lift an object there is an anticipatory increase in grip force that prevents a grasped object from slipping. When an object is pulled from our grasp by an external force, a reflexive modulation in grip force prevents slippage. Here we examine how external perturbations to a grasped object influence anticipatory grip force during object manipulation using a bimanual task, with each hand holding a computer-controlled object. Subjects were instructed to maintain the position of the object held in the right hand. Loading was applied to this restrained object: either self-generated by the action of their left hand or externally generated by a motor. The magnitude of the grip force response to self-generated loading increased after the object was loaded, and the latency of this response remained predictive of load force. This implies that external and self-generated loading increase the anticipatory grip force response. Unlinked trials, where the subject's moved their left hand but no loading was generated on the right-hand object were used to assess the presence of purely predictive control of grip force. External loading soon after self-generated loading maintained an existing predictive response once the linkage between the subject's action and object loading had been removed. However, external loading had no influence as the existing prediction decays. Therefore, the predictive grip force response during object manipulation can be significantly modified by object loading from an external source.

When the position of our body [7] or a hand held object [8] is compromised through perturbations, our motor system acts to maintain stability. Perturbations to postural stability can occur through either the imposition of external loading [2] or our own actions [3].

If we self-generate a perturbation, for instance when one hand pulls on an object held in the other, efference copy of our motor command enables us to anticipate the consequences of our action [3–5]. Therefore, there is a predictive increase in grip force that prevents the grasped object from slipping. If a perturbation to an object is caused by loading from an external source, for instance when someone pulls on the object that we are holding, our motor system will instead reflexively adjust grip force to compensate for the alteration in load force [2]. Predictive adjustments of grip force are characterized by faster latencies and lower magnitudes than the reflexive grip force response [8]. The repeated experience of external loading on a grasped object, can also lead to the development of an expectation of a perturbation [9,10]. Both of these forms of prediction involve the development of associations, either between our action and its consequences [5,1] or between holding an object and it moving.

It is not understood how previous external loading influences the predictive grip force that occurs to self-generated loading. In this study, subjects performed a bi-manual task, holding a computer-controlled object in each hand (Fig. 1). Different loadings occurred on the right-hand object, either self or externally generated, and subjects were instructed to maintain this object's position. On self-generated trials, subjects pulled on the left hand object. When the objects are linked together by computer control, this action led to equal and opposite loading on the right-hand object. Previous studies have found predictive grip force develops, preventing the restrained object from slippage [12–14]. Grip force slightly lags the load force and could reflect a composite of a precisely timed prediction and a delayed reflexive grip force. By removing the linkage between action and consequence on unlinked trials we have been able to assess the purely predictive component as no loading is applied to the object.

There are two questions this paper aims to address using this paradigm. First, in the ‘Development of Prediction’ experiment, whether previous external loading leads to an increase in the magnitude of grip force to self-generated loading. Second, in the ‘Decay of Prediction’ experiment, whether previous external loading influences the predictive or the reflexive component of the grip force response.

Twelve right-handed subjects (3 males, 9 females), naïve to the research aims participated in the study. The study, carried out according to the declaration of Helsinki, was approved by the local ethics committee. Each subject provided written informed consent.

Six subjects participated in the each experiment (Development of Prediction, Decay of Prediction). None reported any sensory or motor deficits.

Subjects held separate cylindrical objects with each hand using a precision, thumb-index finger grip (see Fig. 1). The cylinders had two parallel suede-covered grip surfaces (diameter 30 mm, separation of 40 mm). A 6-axis force transducer (Nano, ATI Inc.) was embedded in each cylindrical object with the mass of the transducer centered midway between the surfaces. The total mass of each object was 50 g. The force transducer allowed three translational forces to be measured with an accuracy of 0.05 N including cross-talk. Both objects were attached by an aluminium rod (length 50 mm) to a torque motor under robotic control. Vertical forces could be generated on the object in a computer-controlled fashion with an update rate of 1000 Hz.

During each trial the subject was instructed to maintain the position of the right-hand object. The start of a self-generated trial was signalled by a tone approximately every 3 s. On hearing this tone, subjects made a short upward pulse with their left hand. The position of the left hand object was displayed as a scrolling trace on a computer monitor and the required amplitude of 6 mm was displayed as a constant horizontal line on the scrolling trace. The consequence of this action on the right-hand object was either linked or unlinked. To ensure the load forces to the left hand were the same during linked and unlinked trials, the motion of the left hand acted against a simulated stiff spring of 1 N/mm attached to the left hand object's initial position. On linked trials, a load force equal and opposite to load force on the left hand object, was applied to the right-hand object. On unlinked trials, no load force was generated on the right-hand object. To prevent any prior knowledge of whether the trial was linked or unlinked, based on cues from accidental small movements of the left hand, the force on the right object was zero until the tone in all trials.

In external trials, no tone sounded and the right-hand motor generated a load force pulse on the right-hand object. The temporal profile of the external load force pulse was chosen to be smooth and bell shaped so that it matched the profile produced by the subjects on linked trials. Specifically, the force profile had the same shape as the velocity profile of a minimum jerk movement [6]. The duration of the force profile was 200 ms and its maximum amplitude was 6 N. Therefore, a peak load force was generated on the fingers of the right hand of 6 N.

Here we examine how self-generated and external loading influences grip force magnitude to subsequent self-generated trials. A control condition assessed how external loading altered grip baseline. Conditions (Linked–Unlinked, Linked–External and External–Null) were performed in a counter-balanced order across the six subjects. Each condition was a series of 200 trials in which each trial was pseudo-randomly chosen to be one of two different possible trial types with equal likelihood [14]. First, in condition Linked–Unlinked, the influence of self-generated loading on prediction was examined. All trials were self-generated, with two possible consequences of this action; linked or unlinked trials. On unlinked trials the coupling between the action of the subject's left hand and a loading on the right hand was removed, and the two objects behaved independently. To assess the influence of external loading on grip force to self-generated loading, condition Linked–External trials presented subjects with “Linked” or “External” trials. To examine the effect of external loading on baseline grip force, condition External–Null was included. Trials were either External or Null. On null trials nothing happened; no load force was applied to the object and subjects were not required to generate a load force on the object by the action of their left hand.

The second experiment examined the influence of external loading on the purely predictive component of grip force. Six subjects participated using a method similar to an earlier study [12]. Subjects performed 33 batches of trials. Each batch had a 3 s spaced sequence of trials that comprised three successive linked trials, where a grip force prediction would be expected to develop, followed by between 3 and 7 unlinked trials, where this prediction would decay. A single external pulse was introduced after one of the unlinked trials (timing midway in timing between two normal trials). In total there were 270 trials. Grip force on unlinked trials must be predictive as there is no actual loading of the object [12,14]. If external loading increases the reflexive component of grip force no effect of external loading would be expected on unlinked trials. Alternatively, if external loading influences prediction, grip force modulation would increase on the following unlinked trial.

For all trials, in both experiments, the position of both hands, grip and load forces on the object in the right hand were recorded at 200 Hz. To quantify the magnitude and timing of anticipatory grip force; the amplitude and timing of the peak grip force modulation for the right hand was found for each trial. For statistical analysis grip force modulation was taken as the difference between the grip force (grip force within a 300 ms window on either side of the maximum load force to the right-hand object) and the baseline grip (average value of the grip force in the first 100 ms of each trial). This measure of modulation of grip force, rather than actual grip force was determined as it is increased modulation that is the characteristic feature of predictive grip responses [8]. The grip force lag was calculated as the difference between the time of the peak grip force and the time of peak load force (with negative values indicating grip force precedes load force). On unlinked trials when no load force was generated on the right-hand object, and linked trials (in Condition Linked–Unlinked only), peak grip force modulation and lag was found in reference to peak left hand discursion in both experiments. The point of peak left-hand discursion corresponds the time of peak load force on linked trials. MANOVAs were used to compare the grip force response on the two trial types in each condition (Linked–Unlinked, Linked–External and External–Null) and to compare the magnitude and lag of the grip force response for each condition dependent on the previous trial experience. To compare the response on the different trial types a one way repeated measures MANOVA was used with a factor trial type (3 levels: linked, unlinked, external). To examine the effect of external loading interspersed in the unlinked decay series a MANOVA with factors of Ago (1–4 post linkage) and History (2 levels: Previous external, Previous Unlinked). When appropriate, Tukey HSD post hoc tests were used.

On linked trials, average peak grip force modulation (grip force change from baseline) was 6.7 N, and lagged the peak load force by 6.9 ms. Consistent with previous studies [12,14], peak grip force modulation on unlinked trials was significantly smaller than linked trials at 4.6 N (*F*(1,5) = 25.4, *p* < 0.005) and occurred significantly earlier than linked trials; in advance of the peak load by 13.9 ms (*F*(1,5) = 25.43, *p* < 0.005) [12]. There was no difference in mean grip force baseline (2.5 N for both trial types).

The influence of loading was examined by comparing grip force histories. Consistent with previous work [14], linked trials (acting as single object) increased the magnitude of grip force prediction on subsequent unlinked trials (acting as independent objects), by 1.2 N, _L_U trial magnitude of 5.2 N compared with the magnitude after consecutive unlinked trials (_U_U trial, 4.0 N) (*p* < 0.0005) (Fig. 2a and d). There was no influence on the latency of the unlinked trial dependent on trial history, with peak grip force preceding the peak left hand position, the position that would have reflected the maximum loading if the objects had been linked to simulate a single object, by 14 ms in both cases.

There was no significant effect of the occurrence of an unlinked trial on the grip force magnitude of subsequent linked trials (Fig. 2e). However, the grip force on linked trials with a prior unlinked trial (_U_L) lagged the peak of the load force by 10 ms, significantly longer than the latency where there were consecutive linked trials (_L_L), that lagged the peak load by 3.6 ms (*p* < 0.01).

On linked trials, average peak grip force modulation was 10.8 N compared with a mean modulation of 11.3 N on external trials. On linked trials all subjects’ grip force was predictive of load force (mean 21.7 ms) compared with external trials where the grip force lagged peak load by 54.3 ms, a latency associated with reflexive grip force responses (*F*(1,5) = 36.36, *p* < 0.01). Baseline on external trials in this condition (3.8 N) was significantly higher than on linked trials (3.4 N, *p* < 0.05).

The magnitude of the mean peak grip force modulation on linked trials was significantly greater than in condition Linked–Unlinked (6.7 N): *F*(1,5) = 18.3, *p* < 0.05. This implies external loading increases grip force magnitude on self-generated loading. The mean magnitude of the grip force baseline (3.6 N) was elevated compared the Linked–Unlinked and External–Null conditions, both *p* < 0.01).

Grip force modulation was significantly higher in linked trials with previous external loading (_E_L): 11.2 N compared with 10.2 N (*F*(1,5) = 8.3, *p* < 0.05) than when there were consecutive linked trials (_L_L, Fig. 2b and g). Therefore, external loading increases grip force magnitude on subsequent linked trials without an increase in the latency of the grip force response that would be associated with a stronger reflexive component. There was no significant difference in the magnitude or latency of the grip force modulation on external trials dependent on trial history (Fig. 2f). There was no significant effect of trial history on grip force baseline in either external or linked trials.

Average peak grip force modulation during the Null trials was 0.7 N was significantly smaller than the average peak grip force modulation during the external trial of 11.6 N (*F*(1,5) = 14.6, *p* < 0.01). The lag of the external trial was 59 ms, a latency indicating a reflexive grip force response and not significantly different from the lag of the external trial in condition Linked–External.

There was no significant effect of the trial history on the external trials (Fig. 2i) but there were effects on Null trials (Fig. 2c and h). The grip force peak on Null trials was significantly higher if the trial followed an external trial, _E_N than if it followed a Null trial, _N_N (4.0 N compared with 3.4 N (*F*(1,5) = 7.758, *p* < 0.05), however similar to previous studies, this peak is minimal and the timing of this response on Null trials was inconsistent [13].

Therefore, both self-generated and external loading increased grip force response. Latency of the grip force response on _E_L trials remained predictive, indicating that the predictive component may be influenced. However, this cannot be determined from this experiment. The next experiment includes unlinked trials, where the loading to the object is absent, and therefore grip force response on these trials must be predictive. The effect of external loading on prediction can then be examined.

This experiment examined how external loading affected the predictive component of grip force modulation. There were three successive linked trials over which grip force prediction developed in each batch. Magnitude of grip force modulation increased from an average of 7 N on the first linked trial to 8 N on the third linked trial (*F*(2,10) = 5.6, *p* < 0.05). Timing of the grip force peak did not change significantly over the three trials (average lag of 1.8 ms).

The linked trials were followed by 3–7 unlinked trials (Fig. 3a–c). Grip force profiles and modulation on successive unlinked trials where loading occurs after the first unlinked (Fig. 3a and c) and after the fifth unlinked (Fig. 3b and c). In the absence of external loading there was a significant decrease in grip force modulation between the first and second unlinked trials of 0.8 N (Fig. 3d first point on _U_U, *p* < 0.05). This is consistent with a previous study [12]. However, if an external loading trial was inserted between the first and second unlinked trial, predictive grip force significantly increased by 2.1 N (Fig. 3d first point on _UE_U, *p* < 0.05). There were no significant changes in grip force modulation when the external loading occurred later in the series of unlinked trials (Fig. 3d points 2–4). Therefore, external loading only modifies prediction when there is a strong association between action and consequence.

Baseline effects of external loading on unlinked trials were similar throughout the unlinked series with no significant differences due to a prior external loading.

This study, using a bi-manual virtual object task, confirmed previous findings that predictive grip force develops when a computer-controlled linkage is introduced between the action of one hand and the consequence of a loading on the other. This linkage simulates the situation of a single object held between two hands. The grip force response to self-generated loadings is thought to comprise of both predictive and reflexive components. Here we examined the situation where only the restrained object was externally loaded. On subsequent self-generated loadings the grip force increased. This increase could be due to the gain changes in either the reflexive or the predictive component of the grip force response.

In the ‘Development of Prediction’, prior self-generated (_L_U) and external loading (_E_L) increased grip force response. Moreover, external loading increased grip force modulation on subsequent linked trials (_E_L) more than consecutive linked trials (_L_L). As grip force latency remained too fast to be reflexive, it is likely that this is an increase in the predictive component of grip force. Additionally, the increase occurs despite external loading being inconsistent with the developed association of a single object being manipulated between two hands. This implies the modulation increase is not due to cognitive knowledge of object properties, suggested in previous studies [9]. Consistent with this, Quaney et al. demonstrated predictions are altered by unrelated voluntary actions. Predictive grip force, modulation as well as baseline, increased when lifting an object of known weight when an isolated pinch prior to lift had been performed. This effect occurred regardless of whether the lifting or the non-lifting hand pinched [11]. Here we demonstrate that an unrelated loading, with a similar temporal profile to the self-generated loading, increases the subsequent predictive response to self-generated loading.

The second experiment examined the influence of external loading on the predictive component of grip force. Previously, a predictive grip force response has been shown to develop after three linked trials, and then slowly decay over a series of unlinked trials [12]. As no loading occurs on unlinked trials, they can assess the presence of predictive control. Here we used the same design, but with an external loading inserted at a randomized point within the decay series. Unlinked trials were used to examine whether external loading influences the grip force prediction or alters the gain of the reflexive response. If the reflexive component were affected, no influence of external loading would be expected on these trials. When the external loading occurred after just one unlinked trial, magnitude of grip force modulation (but not grip force baseline) on the second unlinked trial increased significantly. However, if external loading occurred later in the series of unlinked trials there was no effect. Therefore, grip force prediction has to be of sufficient strength for the gain to be increased by external loading. Therefore, initially the external loading has a similar influence to a further linked trial. However, this is only when there is an existing strong prediction.

The differential effect of external loading on grip force prediction across the series of unlinked trials suggests the effect is not due to increased vigilance of object slippage enhancing current prediction. If external loading enhanced current prediction, the gain increase should occur throughout the decay series. Vigilance does however lead to an elevation of mean grip force baseline in condition Linked–External in the first experiment. However, effects of grip force baseline dependent on trial history were not significant in either experiment.

To conclude, this study shows that prior self-generated or external loading increases the grip force response to subsequent self-generated loading. Surprisingly, external loading alters the purely predictive component of grip force. However, this effect of external loading only occurred when there was a strongly developed association of a single object being manipulated between the two hands.

## Figures and Tables

**Fig. 1:**
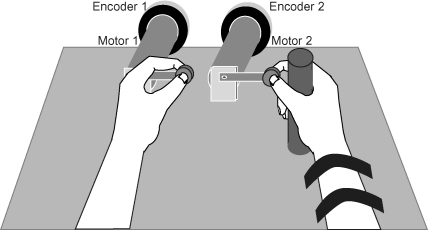
Schematic diagram of the apparatus used to create objects with novel dynamic relations. Each hand held a computer-controlled object with a force-transducer embedded and attached to a torque motor. The subject was required to pull on the fixed left object and to maintain the position of the object held in the right hand. The objects could be either “linked”, so they acted together, or “unlinked”, so that they acted as two independent objects. On external trials, the motors pulled on the right-hand object.

**Fig. 2:**
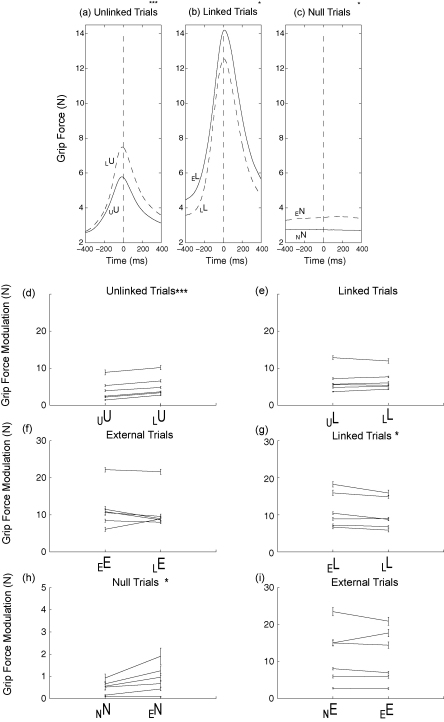
Average across the subjects’ mean grip force (N) for trials with a significant influence of trial history, where _L_U means the modulation of unlinked trials which follows linkage (and similarly for other trial types) (*N* = 6). (a and b) Condition Linked–Unlinked; (c and d) condition Linked–External; (e and f) condition External–Null. The vertical dashed line is the time of peak load force (or for the unlinked trials time of expected peak load force). Subjects’ mean grip force modulation (N) for each of the three conditions dependent on the previously experienced trial with all trial types shown (a and b) condition Linked–Unlinked c–d) condition Linked–External. (e and f) Condition Null–External. Error bars show S.E. individual means (*N* = 6) ((*) significant at *p* < 0.05; (***) significant at *p* < 0.005).

**Fig. 3:**
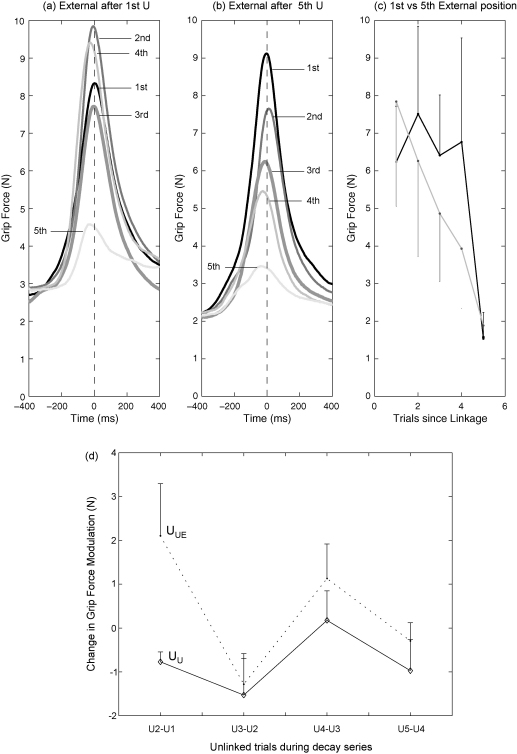
Average across subject's mean grip force on successive unlinked trials in a series where external loading was after (a) first unlinked trial (*N* = 6); (b) fifth unlinked trial (*N* = 5) c.f. previous work [12]. (c) Average across subject's mean grip force modulation (same data series as 3a and 3b) External after first unlinked (dark), after fifth unlinked (pale). (d) Average across subject's mean change in (d) grip force modulation (N). External trial present between the pair (dashed line). No external trial present between the pair (solid line). Error bars show S.E. across subject means.
